# A large-scale repository of spoken narratives in French, German and Spanish from Cantonese-speaking learners

**DOI:** 10.1038/s41597-023-02090-6

**Published:** 2023-04-05

**Authors:** Xin Kang, Virginia Yip, Stephen Matthews, Patrick C. M. Wong

**Affiliations:** 1 Department of Linguistics and Modern Languages, The Chinese University of Hong Kong, Shatin, Hong Kong SAR China; 2 Brain and Mind Institute, The Chinese University of Hong Kong, Shatin, Hong Kong SAR China; 3 Research Center for Language, Cognition and Language Application, Chongqing University, Chongqing, China; 4 School of Foreign Languages and Cultures, Chongqing University, Chongqing, China; 5 Childhood Bilingualism Research Center, The Chinese University of Hong Kong, Shatin, Hong Kong SAR China; 6 Department of Linguistics, The University of Hong Kong, Shatin, Hong Kong SAR China

**Keywords:** Interdisciplinary studies, Human behaviour

## Abstract

Interdisciplinary research on foreign language learning has important implications for learning and education. In this paper, we present the Repository of Third Language (L3) Spoken Narratives from Modern Language Learners in Hong Kong (L3HK Repository). This database contains 906 audio recordings and annotated transcripts of spoken narratives in French, German, and Spanish that were elicited from Cantonese-speaking (L1) young adults using a wordless picture book, “*Frog, Where Are You?*”. All participants spoke English as the second language (L2) and learned the target language as a third language (L3). We collected their demographic information, answers to a motivation questionnaire, parental socioeconomic status, and music background. Furthermore, for a subset of participants, we collected their L1 and L2 proficiency scores and additional experimental data on working memory and music perception. This database is valuable for examining cross-sectional changes in foreign language learning. The extensive data on phenotypes provide opportunities to explore learner-internal and learner-external factors in foreign language learning outcomes. These data may also be helpful for those who work on speech recognition.

## Background & Summary

Storytelling is a universal human activity that has existed from the Stone Age to the present^1,2^. The ability to tell a story or narrate involves using social-cognitive knowledge and complex language. Systematic analysis of storytelling has been used to quantify the language abilities of typical and special populations^3–5^, the psychological and emotional status of individuals^6^, and clinical investigation of special populations^7–9^.

However, existing open datasets on the storytelling of language learners have some limitations. Most of these databases have a limited sample size. For example, in the TalkBank^10^, a major open-sourced collection of corpora with second or foreign language learners, the sample sizes of its collections vary from 1 to 600. In addition, most of these databases do not include detailed background information about learners, such as their social-economic status^11^, music background^12–14^, and language learning motivation^15^. Furthermore, in a world in which learning languages other than English is common, it is vital for researchers to have access to databases of foreign language learners of different languages.

This paper introduces the Repository of Third Language (L3) Spoken Narratives from Modern Language Learners in Hong Kong (L3HK Repository), which contains 906 audio recordings and transcripts of storytelling in French, German, and Spanish and detailed phenotypes of young Cantonese-speaking adults who learned these languages as the third language (L3) in Hong Kong. The recordings were elicited from Mercer Mayer’s wordless picture book *Frog, Where Are You?*^16^. The book depicts a missing frog, a boy and his dog who set out on a journey to find it. Trained native speakers of the target languages transcribed the audio recordings in the Codes for the Human Analysis of Transcripts (CHAT) format, and we morphosyntactically tagged these transcripts using the Computerized Language Analysis (CLAN) program^17^. This repository also contains information on the backgrounds of learners, including demographic information (e.g., gender, age), motivation for language learning, classroom performance, and picture naming as measures of language access^18^. Furthermore, we collected the first (L1) and second (L2) language proficiency scores and experimental data on working memory and music perception for a subset of participants.

With its cross-sectional design and relatively large sample size, this repository will facilitate large-scale quantitative investigations into foreign language learning theories, especially systematic studies of participants with a homogeneous background learning these languages. Given that the repository also contains multiple measures of foreign language proficiency for each individual, it is possible to directly test for associations between proficiency measures and aspects of language production at the word, sentence, and discourse levels. The acoustic and phonological characteristics of speech samples, such as suprasegmental measures, can also be useful in studies on language abilities^19^. These cross-sectional data will allow testing theories of foreign language learning. It will also enable the testing of predictive models on foreign language learning outcomes. Furthermore, it can examine the relationship between individual differences in foreign language skills, learner-internal and learner-external factors. The results of these studies will be useful in identifying learners who may be slow or fast in learning foreign languages. Thus, these data may guide the teaching and learning of foreign languages theoretically and empirically. Importantly, we expect this repository to contribute to the reproducibility and replicability of foreign language learning studies. In addition, researchers working on speech recognition may also find the repository valuable for identifying foreign-accented French, German, and Spanish speeches.

## Methods

### Participants

The database includes 906 participants (672 females) between 18 and 25 years of age (Mean = 19.98, SD = 1.27) recruited from a research university in Hong Kong through mass emails and advertisements in their language classes after obtaining permission from the class teachers. See Table 1 for the demographics of participants.

**Table 1:** Demographics of participants.

Variable	Description	Language		
		French	German	Spanish
**Class level**	**Level II**	166 (57%)	116 (47%)	196 (53%)
	**Level III**	32 (11%)	20 (8%)	33 (9%)
	**Level IV**	72 (24%)	64 (26%)	105 (29%)
	**Level V**	11 (4%)	22 (9%)	15 (4%)
	**Level VI**	11 (4%)	23 (10%)	20 (5%)
	**Total**	292	245	369
**Self-rated level**	**Beginner**	41 (14%)	38 (16%)	57 (15%)
	**Post-beginner**	161 (55%)	119 (49%)	183 (49%)
	**Lower Intermediate**	72 (25%)	74 (30%)	111 (30%)
	**Intermediate**	10 (3%)	13 (4%)	17 (5%)
	**Upper Intermediate**	5 (2%)	1 (1%)	1 (1%)
	**Missing data**	3 (1%)	0	0
**Gender**	**Female**	222 (76%)	169 (69%)	281 (76%)
	**Male**	70 (24%)	76 (31%)	88 (24%)
**Age**	**Mean [range]**	20 [18–25]	20 [18–25]	20 [18–24]
**Non-verbal IQ**	**Mean (SD)**	109 (10)	108 (11)	107 (10)
**Music training**	**Yes**	248 (85%)	196 (80%)	289 (78%)
	**No**	40 (14%)	46 (19%)	79 (21%)
	**Missing data**	4 (1%)	3 (1%)	1 (1%)
**The onset age of music training**	**Mean [range]**	8 [1–20]	8 [1–20]	8 [1–20]
**Years of music training**	**Mean [SD]**	6 (5)	6 (5)	5 (5)
**Family SES**	**Mean (SD)**	38 (16)	36 (16)	36 (15)

All participants were native speakers of Hong Kong Cantonese, a variety of spoken Chinese, used by 88.2% of the population in Hong Kong^20^. They started learning English as a second language (L2) from a young age (less than 5 years old). They had varied experiences learning the target language before entering university, with an average initial age of exposure being around 18 years old. Participants learned French (n = 292), German (n = 245), or Spanish (n = 369) as a third language. These courses were taught mostly by native speakers of the target languages in a classroom setting at the university. Classes took place once a week and each session lasted about 3 hours. In total, participants had 36–42 hours of L3 classes per semester. Class levels were classified based on the teaching goals. Level I refers to the beginning level with no prior experience of the target language required. The highest level of classes is Level VI. Participants were enrolled in a higher level class the next term (e.g., Level II) after they passed the exams at the end of each semester. They all had a nonverbal IQ of at least 85, as measured by the Test of Nonverbal Intelligence, Fourth Edition (TONI-4)^21^. They had no self-reported hearing, psychiatric, or neurological disorders. All participants underwent a hearing test that was carried out for frequencies of 500, 1k, 2k and 4k Hz at 30 dB HL in a soundproof booth. The research protocol was approved by the Joint Chinese University of Hong Kong – New Territories East Cluster Clinical Research Ethics Committee. Written informed consent was obtained from each participant for joining this study and sharing their data anonymously for the purpose of teaching and research.

### Narrative production

Participants were individually tested in a quiet room. The stimuli were black-and-white pictures from the children’s wordless storybook “*Frog, Where Are You*?” by Mercer Mayer^16^. Participants were instructed to tell the story in the third language and use the picture book as a visual prompt. A microphone recorded their narrative production using the Praat program^22^ on a laptop computer.

#### Transcription

Transcription of the recordings was prepared according to the CHAT conventions of the Child Language Data Exchange System (CHILDES)^17^ by native speakers of the target languages. The transcribers were trained to use the CHAT format following the procedures described by MacWhinney^17^ and the latest versions of the CHAT manual^23^. The first transcriber transcribed all recordings verbatim, while the second transcriber transcribed at least 10% of the files of the first transcriber directly from the recordings. The second transcriber also checked all the transcription and coded errors at the word (e.g., inflectional errors) and sentence levels (e.g., incomplete sentences). The transcription of the first transcriber was used for data analysis unless the discrepancy between two transcripts exceeded 85% (after excluding punctuations). In this case, the two transcribers discussed the differences and agreed on the final version.

#### Coding

The morphosyntactic annotation was automatically coded using the MOR and POST programs in the CLAN command window after installing the morphosyntactic dictionaries of the target languages^24–26^. On average, the MOR command auto-tagged 92.5% of the words in three languages.

### Pronunciation ratings

Another group of native speakers of the target languages with no/very-limited exposure to Cantonese rated how native-like the learners’ pronunciation sounded. Two 20–30 second excerpts per recording were taken from the beginning and the end, excluding any initial pauses or false starts. Two counterbalanced lists were created for each language. To ensure that each participant could complete the rating task in roughly the same estimated amount of time, each list was divided into four sub-questionnaires for French (N = 292) and German (N = 245) and six for Spanish (N = 369). Each sub-questionnaire contained a similar number of trials and could be completed in around 30 minutes. Sixteen native speakers were recruited to rate the recordings in every sub-questionnaire using a 9-point Visual Analog Scale^27^ from 1 (not native at all) to 9 (very native-like). The recordings were presented to them in randomized order through Qualtrics.com. We averaged the scores among the raters to give separate L3 pronunciation ratings for the two excerpts from the beginning and end of the narrative. We found that the ratings for the excerpts at the beginning and the end of audio files were highly correlated (All: *r* = 0.81, *p* < 0.001; French: *r* = 0.86, *p* < 0.001; German: *r* = 0.35, *p* < 0.001; Spanish: *r* = 0.50, *p* < 0.001), suggesting consistency among the raters. The final scores were then averaged across the two excerpts.

### Picture naming

The stimuli were adapted from the body part naming task in the Hawaii Assessment of Language Access (HALA) project^18^. The assumption is that the speed and accuracy of speakers’ access to lexical items indicate the relative strength of languages. Participants were asked to name 31 body parts shown in photographs as quickly as possible (see Table 2). Stimuli were randomly presented to participants using E-prime^28^. Response times were measured from the onset of the photo to the onset of the response in milliseconds (ms). Native speakers of the languages judged whether participants correctly produced the intended words. Only correct trials were included for the calculation of reaction times.

**Table 2:** Test items used in the picture naming task.

High frequency		Medium frequency		Low frequency	
back	ear	arm	cheek	ankle	elbow
eye	face	chin	eyebrow	heel	
fingers	foot	fingernail	forehead		
hand	head	neck	palm		
knee	leg	thumb	toes		
lips	mouth	wrist			
nose	shoulder				
stomach	teeth				
tongue					

### Classroom performance

Participants were evaluated at the end of each academic term. A typical exam consists of speaking, writing, listening, and reading. Three types of exam scores were collected as participants’ classroom performance, including the oral exam score (only speaking), the total exam score (speaking, writing, listening, and reading), and the final score (including the total exam score, homework, and class participation). Participants’ personal information was removed before the data were transferred to the authors for analysis. Standardized z scores were calculated by dividing the difference between the raw and average scores of the learners tested on the same exams by the standard deviation. In this way, we can compare the learners tested at the same class level for each target language.

### Self-reported language levels

Participants reported their self-rated language levels, which have been shown to indicate linguistic ability in a previous study^29^. These self-reported ratings were elicited on a Likert scale from 1 to 5, where 1 was defined as “beginner level” and 5 as “upper intermediate level and above.” See Table 3 for scoring criteria.

**Table 3:** Self-reported proficiency rating criteria.

Score	Criteria
1	**Beginner level** — Able to give simple greetings using set words and phrases. Able to read simple sentences, grasp the gist of short passages, and write a simple sentence in the primary *modern language (ML)*.
2	**Post-Beginner level** — Able to hold a simple conversation such as greeting and introducing someone. Able to read simple materials and write a simple passage in elementary *ML*.
3	**Lower Intermediate level** — Able to converse about familiar daily topics. Able to read materials about familiar everyday topics and write simple letters.
4	**Intermediate level** — Able to converse about general matters of daily life. Able to read general materials related to daily life and write simple passages.
5	**Upper-intermediate level and over**—Able to converse about general matters of daily life and topics of one’s specialty and grasp the gist of lectures and broadcasts. Able to read high-level materials such as newspapers and write about personal ideas.

### Demographic questionnaire

The questionnaire investigates participants’ demographic background, including their date of birth, sex, music training, and parental social-economic status. Participants specified whether they had received music training and listed the style of music or the type of instrument they studied (e.g., piano) and years of training. We also asked about the onset age of music training. In addition, participants provided information on their parents’ education (years and level) and occupational prestige. Family SES was thus measured following the Hollingshead four-factor index of socioeconomic status^30^. Participants’ fluid intelligence was tested using the Test of Nonverbal Intelligence, Fourth Edition (TONI-4)^21^.

### Motivation questionnaire

The Modern Language (ML) Learner Questionnaire, adapted from Dörnyei and Taguchi^31^, was used to measure motivational and affective factors in the third language learning of participants. The original questionnaire measured 14 factors involved in language learning, including attitudes toward the L3 community, cultural interests, and anxiety. In the first part of the questionnaire, participants were asked to indicate how far they agreed or disagreed with 49 statements (such as “*I have to learn ML because I don’t want to fail the ML course*”) on a 6-point Likert scale ranging from “*Strongly disagree*” to “*Strongly agree*”. In the second part, participants were asked to answer 18 questions (such as “*Do you always look forward to ML classes*?”) on a six-point Likert scale ranging from “*not at all*” to “*very much*”.

### L1 and L2 proficiency

We collected L1 and L2 proficiency as measured by the composite scores of the Chinese (L1) and English (L2) in the Hong Kong Diploma of Secondary Education Examination (HKDSE)^32^ scores from 617 participants. This is the public examination for university admission in Hong Kong, administered by the Hong Kong Examinations and Assessment Authority (HKEAA). L1 and L2 proficiency scores could not be collected from participants admitted to the university using other qualifications (e.g., International Baccalaureate Diploma)^33^. The composite scores were calculated using subtests on reading, writing, speaking, and listening skills on a scale from 1 (lowest) to 7 (highest) for both Chinese and English. An annual calibration exercise was used to ensure that the scores over the years reflect the same performance levels^32^.

### Working memory

293 participants chose to complete an optional running working memory task^14,34^. In this task, participants first heard trials with 3–7 letters (D, F, J, K, L, N, P, Q, R). All letters were normalized for intensity and had the same duration (600 ms). They were presented in randomized order with a 200-msec interstimulus interval. After hearing all letters in a trial, participants were reminded how many letters to report on a response screen. Participants typed the letters in the response box using a keyboard. The letters had to be recalled in forwarding order and in the exact sequence in which they were presented. In total, there were 15 trials covering 75 letters. It took about 15 minutes to complete the task. A point was awarded for each item correctly recalled in the correct position of the trial. For example, if participants heard four letters – F, D, R, N - but responded “D, Q, F, R”, they would receive 0 points, but those who answered “F, D, N, R” would receive 2 points. Participants’ performance was aggregated as a percentage of correct recalls.

### Music perception

305 participants chose to complete additional music perception tasks^14^. The first task is known as the musical pitch perception task. Participants were instructed to listen to a pair of melodies composed on the Western tonal (6 trials), pentatonic (6 trials), and atonal (12 trials) scales and asked to indicate whether the two paired melodies were the same. The duration of each piece was about 11 seconds. The two melodies in each pair were identical in half of the trials. In the other half of the trials, the melodies differed in one note so that one melody had a different pitch from the other melody. In total, there were 24 trials. The task lasted approximately 12 minutes. The second task, known as the rhythm perception task, was similar to the first task except for differences in time (rhythm) instead of musical pitch. The melodies were composed on the Western tonal (6 trials) and pentatonic (6 trials) scales. The duration of each melody was about 11 seconds. In total, there were 12 trials. The task lasted approximately 8 minutes.

## Data Records

This dataset is publicly available under the CC-By Attribution 4.0 International license on Open Science Framework (https://osf.io/djc69/)^35^. All data are anonymous, without any personal information concerning the participants. All data are available in the folder named “*Spoken Narratives from Modern Language Learners in Hong Kong*” which contains two sub-folders, namely “Audio” and “Transcript”.

The “Audio” folder contains audio recordings in .wav format. All files were normalized using peak amplitudes to ensure that the signal peaks of all audio files at 0 dB. This is an automated process that changes the signal by the same fixed amount. This process raises the signal level of all audio files, but it also increases the noise level. Nonetheless, since the content of all audio files remains clear, no further processing, such as noise filtering, was performed. The name of the audio files shows the participant code, the date of data collection, and the target language. For example, “HK-15-024_20160622_spanish.wav” contained the storytelling in Spanish by a participant coded as “HK-15-024” on June 22, 2016. The “Transcript” folder contains transcriptions and annotations of audio files in “.cha” format. Each .cha file has the same name as the corresponding audio file. To open .cha files, CLAN^23,24^ should be used. In addition, demographic information of participants, including age, gender, family SES, music background, and other collected information such as motivation, is provided in a .xlsx file (ngl_scidata_data.xlsx). Each variable is associated with a descriptor that stores its name and other properties, which can be found in the .xlsx file.

## Technical Validation

We performed validation analyses to examine whether narrative production data represented the proficiency of target language learners.

### Features of narrative production

**Fig. 1:**
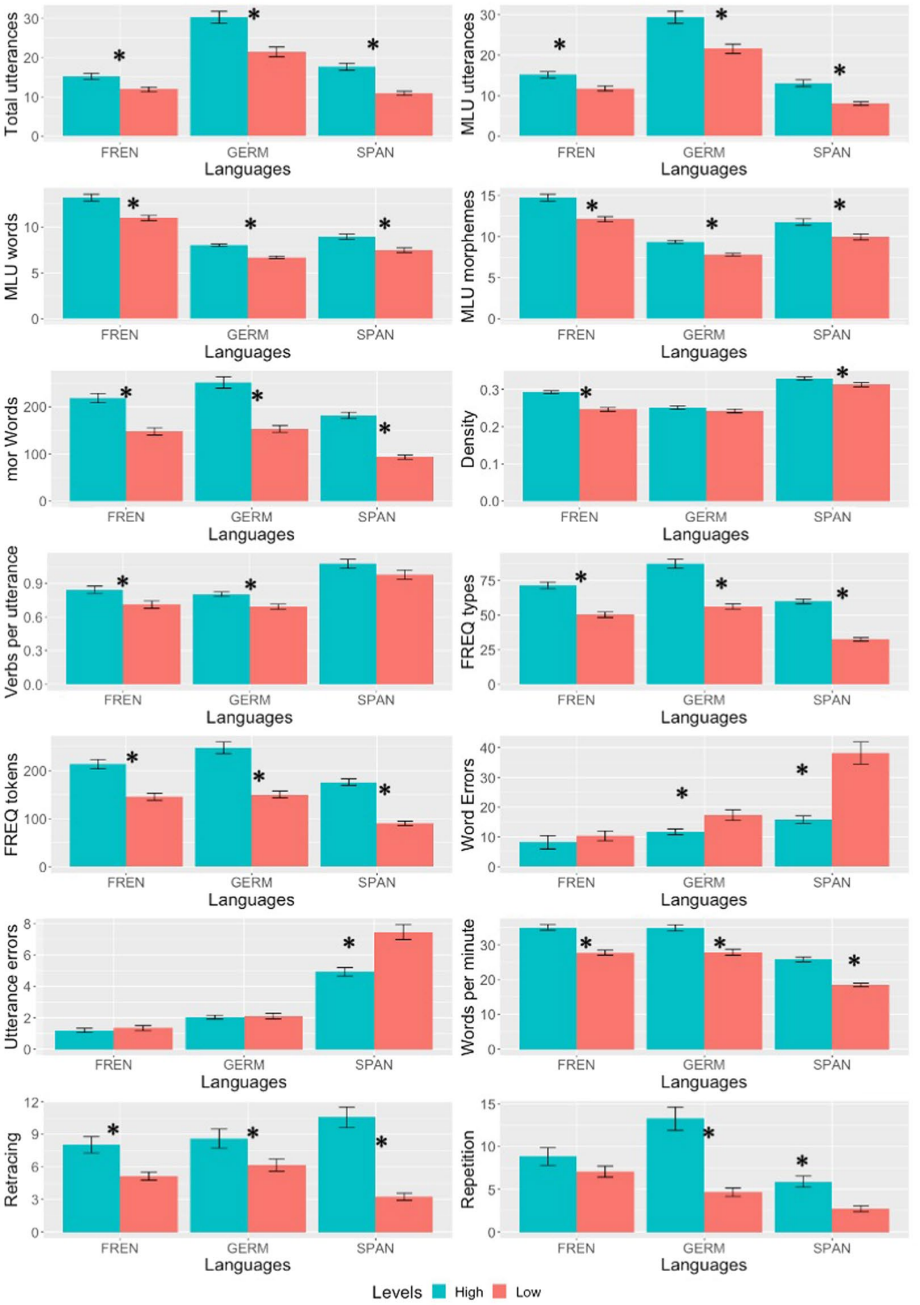
Bar charts of features of narrative production. Learners at low vs high levels of the three target languages showed consistent differences in 9 of 14 narrative characteristics. * indicates that the two groups of learners were statistically different (*p* < 0.05).

Fourteen features of the narrative output were used as outcome measures of target language proficiency, including (1–2) the total number of utterances (all utterances; utterances after excluding unintelligible ones), (3–4) mean length of utterances(MLU) (by wojecrds and by morphemes), (5) total number of words, (6) density, (7) number of verbs per utterance, (8) word types, (9) word tokens, (10) errors at the word level, (11) errors at utterance level, (12) number of words per minute, (13) percentage of retraced words, and (14) percentage of repeated words. The levels of the learners were determined by class levels (from level II to level VI). The low-level learners were enrolled in the Level II class, while the high-level learners were in the Level III class and above. See Table 4 for the results of the t-tests and Fig. 1 for bar charts comparing narrative measures of learners at low vs high levels.

**Table 4:** Results of independent sample t-tests of language levels on features of narrative production.

Features	Target languages			Overall
	French	German	Spanish	
Total utterances	3.34*** (0.40)	4.46*** (0.56)	6.12*** (0.66)	8.07*** (0.55)
MLU utterances	3.39*** (0.40)	4.04*** (0.51)	5.14*** (0.55)	7.01*** (0.48)
MLU words	4.61*** (0.55)	6.78*** (0.86)	3.74*** (0.35)	5.35*** (0.36)
MLU morpheme	4.89*** (0.59)	6.28*** (0.79)	3.28*** (0.59)	5.50*** (0.37)
MOR words	5.45*** (0.66)	6.89*** (0.86)	10.41*** (1.11)	12.43*** (0.84)
Density	7.07*** (0.80)	ns	2.36* (0.24)	4.85*** (0.32)
Verbs per utterance	2.85** (0.33)	3.68** (0.47)	ns	3.58*** (0.24)
Types	6.74*** (0.81)	8.22*** (1.02)	12.84*** (1.36)	14.66*** (0.99)
Tokens	5.39*** (0.65)	6.90*** (0.86)	10.39***(1.11)	12.36*** (0.84)
Word errors	ns	−2.71** (0.36)	−5.71*** (0.57)	5.57*** (0.36)
Utterance errors	ns	ns	−4.58*** (0.46)	−3.75*** (0.24)
Words per minute	6.58*** (0.78)	5.49*** (0.70)	8.48*** (0.90)	10.75*** (0.72)
Retracing	3.37*** (0.10)	2.29* (0.29)	6.93*** (0.76)	7.82*** (0.54)
Repetition	ns	5.86*** (0.72)	4.15*** (0.45)	6.33*** (0.43)

The numbers are *t-values*, Cohen’s D. *** indicates the *p-value* is less than 0.001; ** indicates the *p-value* is less than 0.01; *indicates the *p-value* is less than 0.05; ns indicates that the group differences are not significant (*p* > 0.05).

As shown in Table 4 and Fig. 1, high- and low-level learners of the three languages showed consistent differences in 9 out of 14 characteristics. Cronbach’s alphas for the nine measures and the 14 measures were 0.76 [95% CI 0.75, 0.76] and 0.69 [95% CI 0.68, 0.71], respectively. High-level learners produced longer and more complex utterances with greater lexical diversity. The high-level learners were also more fluent as measured by words per minute but did not differ in accuracy as measured by the percentage of errors.

### Principal component analysis in narrative production

**Fig. 2:**
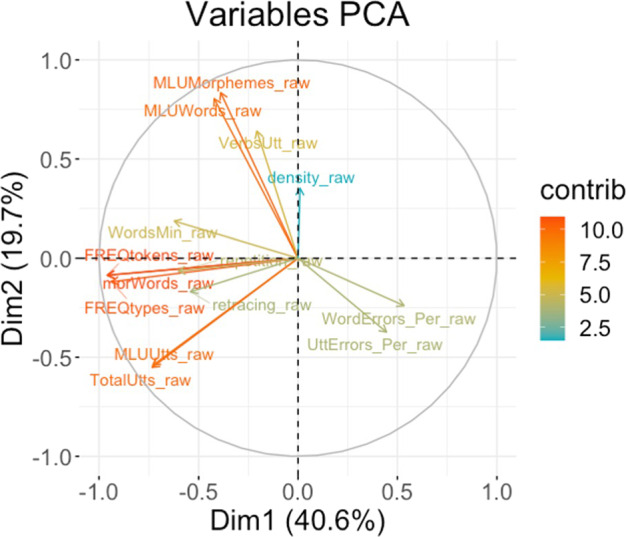
Principal component analysis (PCA) for 14 measures of narrative production. Dimension 1 was indicated by questions related to “length” as indicated by features, including the total number of utterances, the total number of words, and the type of words, while Dimension 2 was labelled as “Content” due to the contribution of mean length of utterances.

**Fig. 3:**
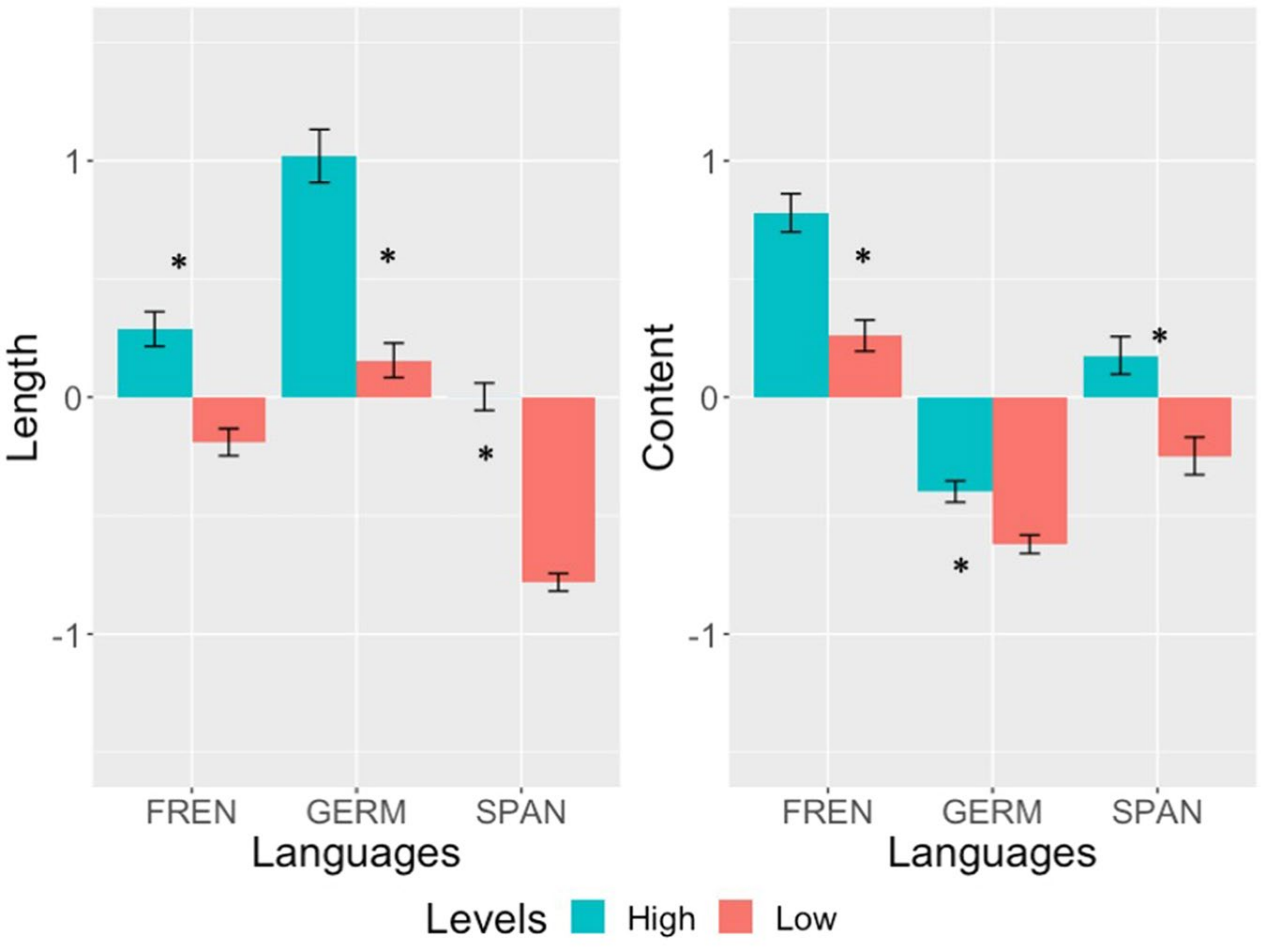
Bar charts of two narrative production components extracted using principal component analysis (PCA). Learners at low versus high levels of the three target languages showed consistent differences in both components. * indicates that the two groups of learners were statistically different (*p* < 0.05).

To reduce the dimensions of these 14 narrative measures, we conducted a principal component analysis (PCA) with varimax rotation. The Kaiser-Meyer-Olkin (KMO) measure verified the sampling adequacy for the analysis with an overall MSA = 0.80, with all variables having an MSA above the cut-off point of.50. Bartlett’s test of sphericity, *χ2* = 1,575, *p* < 0.001, indicated that the correlations between items were sufficiently large for PCA. In this analysis, we extracted two components that explained 60% of the variances, which were named “length” and “content”, based on the factors that contributed to the variances (Fig. 2). Furthermore, we revealed that the two components demonstrated statistically significant differences between learners at the low vs high levels (Fig. 3).

### Relation between narrative production and other measures of language proficiency

**Fig. 4:**
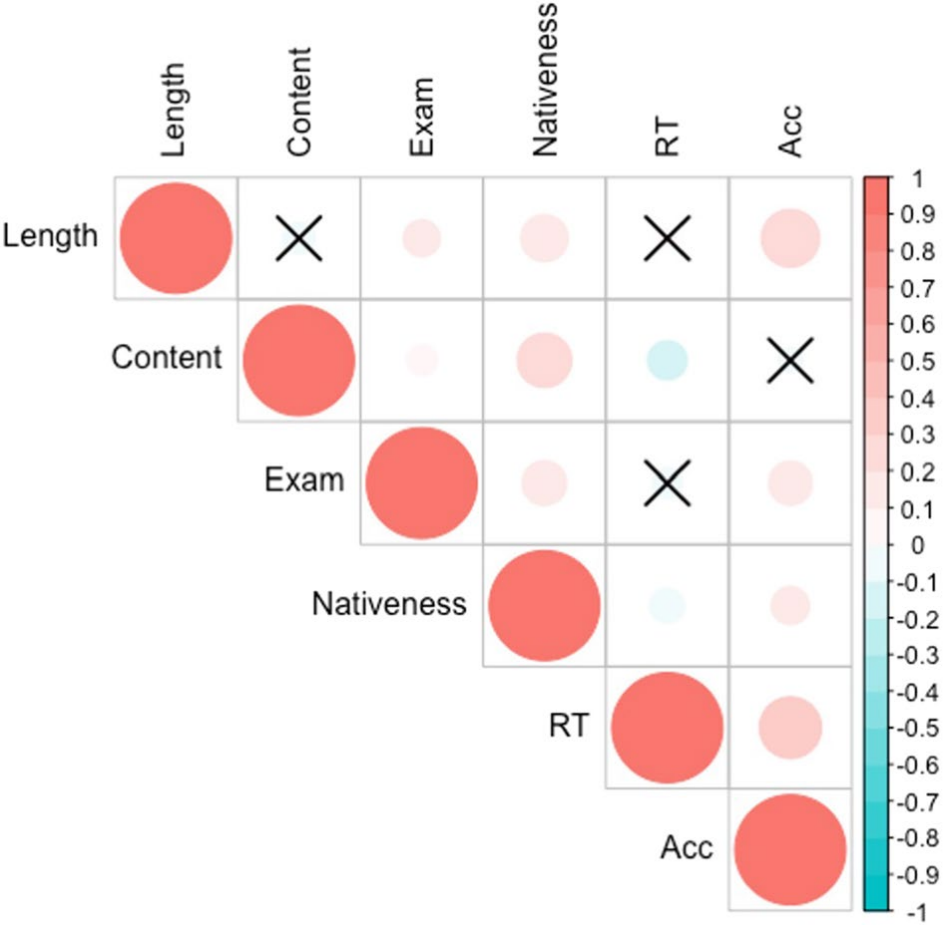
Correlation matrix of two components of the PCA of narrative production and other measures of language proficiency.

Pearson’s correlation coefficients were calculated between the two components of the PCA of narrative production and other language proficiency measures, including accuracy rates and reaction times for picture naming, pronunciation ratings, and exam scores. As shown in Fig. 4, the PCA components of narrative production were correlated with other measures of language proficiency. The length of narrative production was significantly correlated with exam scores (*r* = 0.13, *p* < 0.001), pronunciation ratings (*r* = 0.20, *p* < 0.001), and picture-naming accuracy rates (*r* = 0.33, *p* < 0.001). The ‘Content’ of narrative production was also significantly correlated with exam scores (*r* = 0.08, *p = *0.020), pronunciation ratings (r = 0.27, p < 0.001), and picture name reaction times (*r* = −0.13, *p* < 0.001).

## Usage Notes

All data are publicly available under a CC-By Attribution 4.0 International license. We encourage researchers to use this database for further analysis and publication. Relevant data have been used in previous publications^36,37^.

## Data Availability

The code used to evaluate the quality of the database is in Open Science Framework (https://osf.io/djc69/).
